# Supplementary material for: Effect of bilateral contraction on the ability and accuracy of rapid force production at submaximal force level
Source: PLoS One. 2021 Feb 18;16(2):e0247099. doi: 10.1371/journal.pone.0247099 (PMC7891739; doi:10.1371/journal.pone.0247099)
Supplement: Attachment — Submitted filename: Response to Reviewers.docx [file pone.0247099.s002.docx]

**Response of the Reviewers' comments:**

Thank you very much for your consideration and valuable suggestions for improving my manuscript. I have read the reviewers’ comments very carefully, and have made the following changes according to the comments and suggestions of the reviewers. Moreover, I found mistake, so I corrected the notation of the result (Lines 258-261). The revised page numbers and line numbers are indicated following my response to each of the comments. I hope that the revised manuscript satisfactorily addresses the issues raised by the reviewers. My responses are shown in red text starting with RE.

**Response to Reviewer: 1**

Reviewer #1: Well designed and well presented manuscript. The authors successfully achieved their stated purpose. The authors should replace the bar plots in figures 3-5 with raincloud plots. Below are some links on raincloud plots:
https://wellcomeopenresearch.org/articles/4-63#:~:text=In%20essence%2C%20raincloud%20plots%20combine,error%2C%20such%20as%20a%20boxplot.
https://micahallen.org/2018/03/15/introducing-raincloud-plots/
<https://github.com/RainCloudPlots/RainCloudPlots>

RE: According to the reviewer’s comment, I replaced the bar plots in figures 3-5 with raincloud plots.

**Response to Reviewer: 2**

Reviewer #2: Comments for “Effect of bilateral contraction on the ability and accuracy of rapid force production at submaximal force level”

The purpose of the study was to examine the effects of bilateral contraction on the ability and accuracy of rapid force development (RFD) at the submaximal force level. Eleven subjects performed unilateral (UL) and bilateral (BL) grip contractions at the 30% and 50% of the MVC. No differences were found for RFD between UL and BL contractions in both 30% and 50% MVC. The absolute force error of BL contraction was higher than UL contraction in 30% and 50% of MVC.

One major concern is that the study only examined the force data. If there were other mechanistic variables measured (e.g., rate of EMG rise, etc.), it will be a much-completed nice study. Additionally, one experimental setup regarding the target force (30% and 50% MVC) contraction has flaws and could be influencing the results (see my comments in the later section). Following are my detailed comments:

RE: Although EMG is a useful tool for examining individual muscle activities, there are many muscles related to handgrip strength. Because it is difficult to measured many muscle activities related to handgrip strength, I targeted muscle strength, which is the final output of various muscle activities. Moreover, the present study examined the ability and accuracy of rapid force production in the situation where the submaximal target force levels (30% and 50% MVC). Because the rapid force production below 50% MVC has been confirmed in racket sports, it is not considered that the experimental setup in this study is a flaw.

Stretch R, Buys F, Viljoen G. The kinetics of the drive off the front foot in cricket batting: hand grip force. South African Journal for Research in Sport, Physical Education and Recreation. 1995;18(2):83-93.

Knudson DV, White SC. Forces on the hand in the tennis forehand drive: application of force sensing resistors. Journal of Applied Biomechanics. 1989;5(3):324-31.

Abstract
Please add mean and standard deviation values as well as the p-values when reporting the results. In addition, the author should also report the effect size value.
RE: According to the reviewer’s comment,　I added mean, standard deviation, p-values, and effect size values in the abstract.

Introduction
Lines 55-57, the author missed a few more recent literatures regarding the BLD in peak force and RFD:
Carr, J. C., Bemben, M. G., Black, C. D., Ye, X., & DeFreitas, J. M. (2020). Bilateral deficit in strength but not rapid force during maximal handgrip contractions. European Journal of Sport Science, 1-8.
Ye, X., Miller, W. M., Jeon, S., & Carr, J. C. (2019). Sex comparisons of the bilateral deficit in proximal and distal upper body limb muscles. Human movement science, 64, 329-337.

RE: According to the reviewer’s comment,　I added literatures (Line 60).

Lines 59-60, this statement is not accurate. There are a few recent articles regarding submaximal contractions and accuracy:
Brustio, P. R., Casale, R., Buttacchio, G., Calabrese, M., Bruzzone, M., Rainoldi, A., & Boccia, G. (2019). Relevance of evaluating the rate of torque development in ballistic contractions of submaximal amplitude. Physiological measurement, 40(2), 025002.
Boccia, G., Dardanello, D., Brustio, P. R., Tarperi, C., Festa, L., Zoppirolli, C., ... & Rainoldi, A. (2018). Neuromuscular fatigue does not impair the rate of force development in ballistic contractions of submaximal amplitudes. Frontiers in physiology, 9, 1503.

RE: The literatures introduce by the reviewer are not investigated the effect of bilateral contraction on rapid force performance at low and middle force levels. Therefore, it is not accurate to cite those articles here.

Lines 70-71, only using the force data would still be difficult to have a better understanding of BLD.

RE: According to the reviewer’s comment, the sentence of “Clarifying the effect of bilateral contraction on co-regulating rapid force production ability and accuracy will provide an improved understanding of the BLD.” was deleted.

Lines 72-73, what made the author have such hypothesis? Please comment and clarify.
RE: According to the reviewer’s comment, I changed the sentence of hypothesis. (Lines 74-76)

Methods
Participants: how was the sample size (11) determined?

RE: Sample size was determined from participants who were able to participate during the experiment period.

It was also reported that females and males may have the different bilateral index, especially for the hand muscle (see study: Ye, X., Miller, W. M., Jeon, S., & Carr, J. C. (2019). Sex comparisons of the bilateral deficit in proximal and distal upper body limb muscles. Human movement science, 64, 329-337.). Please justify why you used the mixed sexes for your experiment.

RE: The previous study reported that sex does not influence the magnitude or direction of the bilateral index in the handgrip contraction. Thus, influence of sex on the bilateral index remain unclear. In the present study, I recruited without gender distinction, and did not screening by gender.

Carr, J. C., Bemben, M. G., Black, C. D., Ye, X., & DeFreitas, J. M. (2020). Bilateral deficit in strength but not rapid force during maximal handgrip contractions. European Journal of Sport Science, 1-8.

Lines 121-124, and lines 155-157, it was suggested that targeting the force levels can slow the rate of force production (Gordon, J., Ghez, C. Trajectory control in targeted force impulses. Exp Brain Res 67, 241–252 (1987). https://doi.org/10.1007/BF00248546). How can the author ensure the RFD in the current study was not influenced by the experimental setup?

RE: The purpose of the present study was to clarify the effect of bilateral contraction on the ability and accuracy of rapid force production in the situation where the submaximal target force levels (30% and 50% MVC). The previous study has only given instructions on either “as fast as possible” or “as accurate as possible”. However, in the present study, wherein participants were instructed to grab a dynamometer with 30% and 50% of MVC as fast and precisely as possible. Thus, it is difficult to simply compare to effect of experimental setup on RFD between previous study and present study. Moreover, in the present study, familiarization session is set up to get used to being able to exert rapid force under the submaximal target force levels.

Line 165, please explain the “32-point window”, how long is this window?

RE: Sampling rate of force signal was 1kHz. Thus, “32-point window” is 32ms.

In addition, when calculating RFD, how was the onset of the force signal determined? I could not find the description.

RE: The peak RFD was calculated from the first derivatives of the force signals. Therefore, the peak RFD is calculated without defining of force onset.

Line 170, when calculating force error, what was the time window used in the calculations?

RE: Force errors were determined as the difference between the target force and peak force in each trial. Thus, when calculating force error, the time window was not used in the calculations.

Results and Discussion
Overall, the results are hard to follow.
Line 207, Cohen’s d = 0.11, belongs to trivial size.

RE: According to the reviewer’s comment,　I changed to trivial size. (Line 210)

Please change all the “effect size index” to “effect size”. Also, consider using small, medium, large to describe the effect size.

RE: According to the reviewer’s comment,　I changed “effect size index” to “effect size”. Moreover, I changed the notation of effect size to small, medium, large.

For the discussion, limitations of the experiment should be pointed out.

RE: According to the reviewer’s comment, I added limitations of experiment in the discussion section. (Line 392-401)
