# Supplementary material for: Effect of bilateral contraction on the ability and accuracy of rapid force production at submaximal force level
Source: PLoS One. 2021 Feb 18;16(2):e0247099. doi: 10.1371/journal.pone.0247099 (PMC7891739; doi:10.1371/journal.pone.0247099)
Supplement: Attachment — Submitted filename: Response to Reviewers.docx [file pone.0247099.s003.docx]

**Response of the Reviewers' comments:**

Thank you very much for your consideration and valuable suggestions for improving my manuscript. I have read the reviewers’ comments very carefully, and have made the following changes according to the comments and suggestions of the reviewers. The revised page numbers and line numbers are indicated following my response to each of the comments. I hope that the revised manuscript satisfactorily addresses the issues raised by the reviewers. My responses are shown in red text starting with RE.

**Response to Reviewer: 1**

Reviewer #1: The authors have addressed all concerns. The authors should consider using linear mixed models to analyze individual trials in any future work on this topic, rather than averaging trials and using ANOVA as in the current study.

RE: Thank you for your advice to the future work. I’d like to use your advice as a reference.

**Response to Reviewer: 2**

Reviewer #2: Thank you for addressing my previous comments and revising the manuscript accordingly. Most of the comments were well-addressed.

Previously, I asked "how was the sample size (11) determined?", because I wanted to see if the author had run a power analysis. The current sample size might be underpowered. If this is the case, then it should be mentioned in the Limitation.

RE: According to the reviewer’s comment,　as a result of having calculated statistics power, I confirmed that sample size was small. Thus, I added literatures in Limitation section (Line 393-394).

Previously, I asked "How can the author ensure the RFD in the current study was not influenced by the experimental setup?". I am fine with the response. The author also mentioned "Moreover, in the present study, familiarization session is set up to get used to being able to exert rapid force under the submaximal target force levels." So, please briefly describe what the subjects did during the familiarization.

RE: About familiarization section, explanation is listed in line 108-116.

I saw from Figure 2, that how force error was calculated. What if the actual force was below the target force (the 30% or 50% of the MVC)?

RE: When peak force was lower than the target force, the force error showed negative values. This explanation is listed in line 176-177
